# Responses to digital disinformation as part of hybrid threats: a systematic review on the effects of disinformation and the effectiveness of fact-checking/debunking

**DOI:** 10.12688/openreseurope.14088.1

**Published:** 2022-01-13

**Authors:** Rubén Arcos, Manuel Gertrudix, Cristina Arribas, Monica Cardarilli

**Affiliations:** 1 University Rey Juan Carlos, Móstoles, Madrid, 28933, Spain; 2 European Commission, Joint Research Centre, Ispra, Italy

**Keywords:** Disinformation; Misinformation, Hybrid Threats, Foreign interference, Debunking, Inoculation, Countering disinformation, Systematic Literature Review

## Abstract

The dissemination of purposely deceitful or misleading content to target audiences for political aims or economic purposes constitutes a threat to democratic societies and institutions, and is being increasingly recognized as a major security threat, particularly after evidence and allegations of hostile foreign interference in several countries surfaced in the last five years. Disinformation can also be part of hybrid threat activities. This research paper examines findings on the effects of disinformation and addresses the question of how effective counterstrategies against digital disinformation are, with the aim of assessing the impact of responses such as the exposure and disproval of disinformation content and conspiracy theories. The paper’s objective is to synthetize the main scientific findings on disinformation effects and on the effectiveness of debunking, inoculation, and forewarning strategies against digital disinformation. A mixed methodology is used, combining qualitative interpretive analysis and structured technique for evaluating scientific literature such as a systematic literature review (SLR), following the Preferred Reporting Items for Systematic Reviews and Meta-Analyses (PRISMA) framework.

## Introduction

The hostile influence and interference of state and non-state actors, through the means of communicative interactions that disseminate disinformation to target populations, audio-visual forgeries, and conspiracy theories, constitute a threat to democracies and institutions, harming "collective decision-making” processes ( Price, 2008) in democratic societies. Disinformation-related activities can be part of wider hybrid threats and warfare; information and cyberspace emerge as domains of operations, and battlefields of, information warfare. The target audiences of disinformation implemented as part of hybrid threats are often unaware of these illegitimate uses of digital media by foreign actors and their proxies, and hence prevention and societal resilience building have become cornerstones in countering hybrid threats and disinformation. Assessing the effects of disinformation in societies and the effectiveness of counter responses employed so far is key for honing the counter measures employed by authorities and practitioners, such as fact-checkers and institutional strategic communicators. Having made clear what to do through the development of policies by state authorities, transnational and international organizations, there is also the need to take advantage of lessons learned from academic research and studies on disinformation effects and fact-checking/debunking effectiveness to improve the impact of these countermeasures. That is to say, by developing a better understanding on the effects of disinformation (short and long range effects; and disinformation’s impact on knowledge, attitudes/opinion and behavior) and what fact-checking/debunking practices have proved to be more effective and which others present challenges or can be even counterproductive, practitioners can increase the impact of their strategies and tactics against disinformation.

This paper is hence based on the assumption that, by looking at the main findings from the social and behavioural sciences on the effects of dis- and misinformation, as well as the effectiveness of countermeasures, authorities and professionals can design and establish more effective responses against disinformation as part of hybrid threats. At the same time, research gaps can be identified, providing key research questions for future scholarship and a knowledge base/lessons learnt when tackling disinformation. The article aims to analyse the main findings from academic literature, and relevant reports from security and policy institutions and think tanks, on the effects of disinformation and on the effectiveness of debunking, inoculation and forewarning strategies against digital disinformation. Collecting, analysing, and synthesizing the key findings from the recent literature can provide a solid ground from which to develop further responses. Since the focus of the paper is framed around countering disinformation as part of hybrid threats/warfare, discussion of the results is provided through the lenses of security and communication studies.

There are existing meta-analyses on the effects of disinformation and misinformation, on the one hand, and on the effectiveness of practices as fact-checking and debunking on the other. However, most scholarship on effects is not focused on discussing disinformation activities and campaigns from a hybrid threat perspective. A basic search in Scopus with the combined keywords “hybrid threats” and “disinformation” (TITLE-ABS-KEY [“Hybrid threats” AND disinformation]) provides 8 results (date last run: 11 April 2021).

An important aspect to consider when examining the effects of disinformation and of counter disinformation responses through fact-checking and debunking practices, are theories on the effects of media and how technologies have and are affecting the use of media. As noted by Valkenburg and Oliver “evolving technologies facilitate media “use” well beyond the time boundaries of any single instance of media consumption” (i.e. watching a TV program plus engaging with other users through comments on social media channels), and since the uses of the media are changing by technology there is a need to “revise or develop new ways to conceptualize and measure how individuals now “use” media content and technology” ( Valkenburg & Oliver, 2020, p. 29). Moreover, as noted by Bennet and Livingstone, compared to the era of the traditional mass media, “the current age displays a kaleidoscopic mediascape of television networks, newspapers and magazines (both online and print), YouTube, WikiLeaks, and LiveLeak content, Astroturf think tanks, radical websites spreading disinformation using journalistic formats, Twitter and Facebook among other social media, troll factories, bots, and 4chan discussion threads, among others. Also, important but nearly impossible to study, because of the inaccessibility of data, are Snapchat, Tor-protected websites, and messaging and communication platforms such as WhatsApp, Signal messaging and Voice-over-Internet Protocol, Telegram Messenger, and Proton Mail.” ( Bennet & Livingstone, 2018, p. 129)

In their study of articles published in the Journal of Communication, between 1951 and 2016, Walter *et al.* found that the ten most popular theoretical frameworks or models for the period 2010–2016 were: framing, narrative theory, social identity theory, agenda setting, consistency theory, selective exposure, dual processing models, priming, uses and gratifications, and social cognitive theory ( Walter *et al.*, 2018, p. 436).

The hostile media phenomenon, or effect, was also identified in Walter *et al*.’s analysis (Ibid.). This phenomenon consists of “the tendency for partisans to view media coverage of controversial events as unfairly biased and hostile to the position they advocate” ( Vallone *et al.*, 1985, p. 584). The phenomenon is very relevant for fact-checkers and news organizations since no matter how much neutral identical journalistic reporting and hard news stories are, the same news coverage of events will be perceived as hostile to their own positions by partisans ( Feldman, 2017, p. 2). In our 21 ^st^ Century information environment of digital communication and social networking channels, perceptions of hostile media can lead individuals to reject even independent and balanced journalist reporting or fact-checking organizations and consequently drive people to sources and online communities that reinforce their views and augment the divide between polarized views ( Feldman, 2017, p. 8). This presents a vulnerability that can be utilized by foreign actors aiming to amplify existing socio-political divisions in targeted countries, through campaigns discrediting independent news organizations with different editorial lines and fact-checkers, presenting them as biased or hostile to specific political views. According to this logic, and in the context of the uses of state-funded or affiliated media for information warfare purposes, organizations and professionals conducting the identification, exposure, and debunking of mis- and disinformation would similarly be perceived as unfairly biased by individuals holding partisan views (e.g., domestic individuals and groups at both sides of the political spectrum holding anti-liberalism views and against the model represented by Western liberal democracies). These views can also be strengthened by state-funded media in a deliberate way. Regarding one of the most well-known Russian media organizations, RT, scholars studying its organizational behaviour have argued that the idea “that Western media lies is one of the main elements of RT’s agenda and significantly shapes the ideological foundation of the channel” and that “if there is a story in the U.S. media criticizing the Russian government, RT will respond by criticizing the United States. Whenever Russia is accused of a human rights violation, RT broadcasts stories that suggest that there are comparable cases in the United States” ( Elswah & Howard, 2020, p. 641).

The conceptual framework of hybrid threats, elaborated by the Joint Research Centre (JRC) of the European Commission and the European Centre of Excellence for Countering Hybrid Threats (Hybrid CoE), has introduced the concept of priming as one of the stages in the timeline of hybrid threats activity ( Giannopoulos *et al.*, 2021). According to the conceptual model, this timeline has three different phases (priming, destabilization and coercion) within which the hostile activities (interference influence, operations/campaign, warfare/war) occur, with a strong psychological drive underlying these phases that might overlap; the timeline considers the scalation potential covering short and long-term possibilities. ( Giannopoulos *et al.*, 2021, p. 36). Hybrid threat activities targeting states are conducted in 13 different domains including information, cyber, social, culture, political, diplomacy, infrastructure, legal, military, space, administration, economy, and intelligence. According to the model, in the priming phase the intent of hostile actors “is that the target will voluntarily make harmful choices and decisions” ( Giannopoulos *et al.*, 2021, p. 37). As explained in the JRC/Hybrid CoE’s report, the concept of priming is considered analogous to shaping/conditioning (employed in military and security studies as the preconditioning phase) but with the aim of capturing “the civilian dimension, which is central both as a target and to countering Hybrid Threats” (ibid.). The priming phase is related to “psychological interference” and influence aiming “to prime and by default gain something; information, positioning, testing information, learning or an advantage” (Ibid., p. 38, 40).

In the context of the media, “priming refers to the effects of the content in the media on people’s later behaviour, thoughts, or judgments” ( Ewoldsen & Rhodes, 2020, p. 89). In communication research political priming, or priming effects, addresses “the impact of news coverage on the weight assigned to specific issues in making political judgments” so that when an issue is salient in the “information stream” this will impact its weight when individuals make those judgments ( Iyengar & Simon, 1993, p. 368). As discussed by Van Duyn and Collier, “Individuals are “primed” when information is delivered, stored at the top of one’s memory, and recalled to evaluate subsequent information” ( 2018, p. 4). On priming and media influence, Berkowitz argued that, “how people react to the message they read, hear, or see depends considerably on their interpretations of the message, the ideas they bring with them to the communication, and the thoughts that are activated by it.” ( Berkowitz, 1984, p. 411). 

Regarding activities in the information domain, hostile actors could disseminate disinformation content that later will be recalled by individuals to evaluate posterior information, and this might include evaluations about institutions, political figures’ performances, and others. At the same time, social media platforms can be used by malign actors as a “laboratory” for testing how targeted societies and public opinions might react to messages on issues, identifying potential vulnerabilities through micro-targeting and latter exploiting them in a destabilization phase.

## Methods

This paper is framed under the first cycle of EU-HYBNET project and the project core theme on information and strategic communication. According to the methodological approach of the EU-HYBNET project, there are four core themes to focus on hybrid threats (future trends of hybrid threats, cyber and future technologies, resilient civilians, local and national levels administration, and information and strategic communication). As stated in EU-HYBNET website, “the four project core themes, together with the cycle approach, represent the leading multidisciplinary methodological principles of the project. They create windows for EU-HYBNET to focus on European actors’ awareness, gaps in both understanding and countering and needs for capacity building to strengthen resilience and counter hybrid threats as well to deliver tailor made solutions” ( https://euhybnet.eu/about/). At the start of each project cycle, taking account of vulnerabilities that could be exploited by hybrid threat actors, through a gaps and needs mapping approach what is missing from these perspectives and what capabilities are needed to bridge the gaps are identified.

From this logic, for developing more effective countermeasures against foreign information manipulations and interferences that exploit the current digital information environment and its dynamics, as well as for developing more proactive and effective strategies against disinformation, practitioners in the fields of security, strategic communication and journalism/fact-checking can improve their understanding on mis- and disinformation effects and practice countermeasures that have proven to be effective according to evidence-based research. Hence, the paper aims to collect, analyze and synthetize the main scientific findings on disinformation effects and on the effectiveness of debunking, inoculation, and forewarning practices and strategies against digital disinformation.

More specifically, the main objective of the study was to identify relevant findings from the existing literature that could fall under the following categories and considering cognitive, affective, and/or behavioural effects:

● Disinformation effects and/or impact
● Misinformation effects and/or impact
● Disinformation and misinformation effects and/or impact
● Disinformation as part of hybrid threats
● Fact-checking effectiveness
● Debunking effectiveness
● Measuring exposure to dis- and misinformation

**Figure 1.**
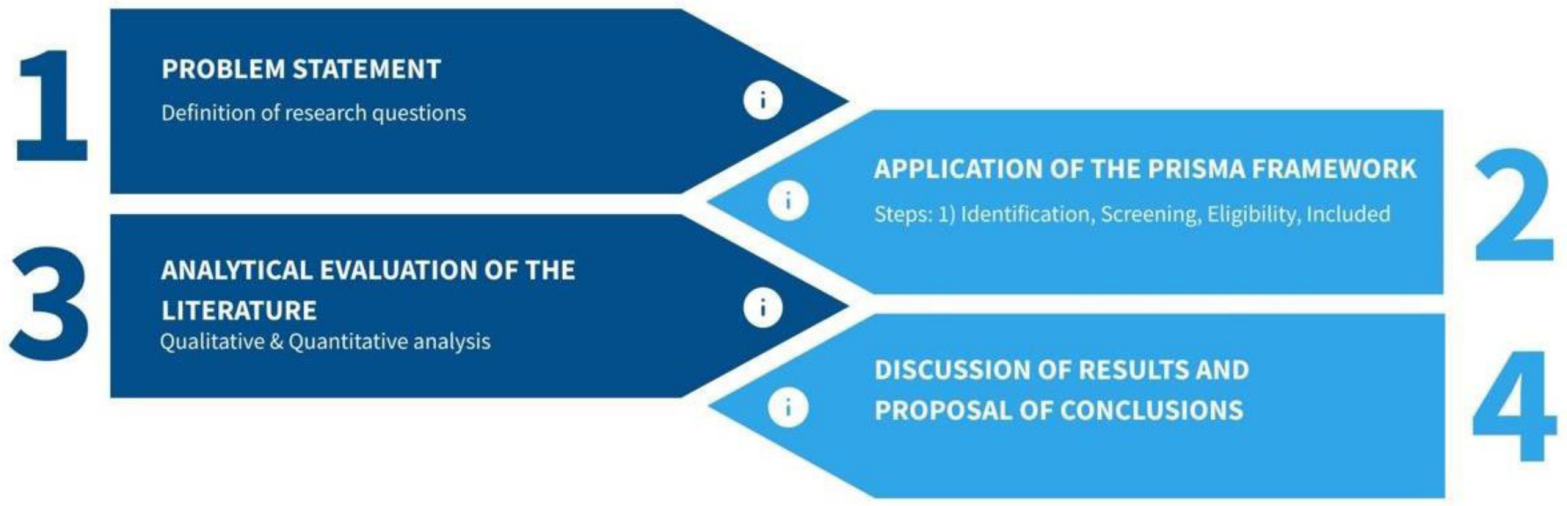
Analysis phases.

A mixed methodology has been used, which combines qualitative interpretive analysis ( Figure 1) as well as the use of a structured technique for evaluating scientific literature such as a systematic literature review (SLR), following the Preferred Reporting Items for Systematic Reviews and Meta-Analyses (PRISMA) framework ( Shamseer *et al.*, 2015).

The scientific studies on the effects of disinformation and fact-checking, and the analysis of findings from this growing literature, is relevant for bridging the knowledge gaps of practitioners involved in addressing and countering information manipulations as part of hybrid threats and foreign interference. This research focuses on the analysis of this literature. 

A combination of qualitative and quantitative methodologies has been used, which allows for the proper integration of the different epistemological approaches of the members of the research team, and the diverse nature of the sources analysed.

The findings first present the results of the PRISMA framework and then the results of the qualitative analysis. The SLR has been carried out following the general PRISMA framework ( Arcos *et al.*, 2021a; Moher *et al.*, 2009) and applying the meta-analysis instrument “EU-HYBNET Meta-Analysis Survey Instrument for Evaluating the Effects of Disinformation and the Effectiveness of counter-responses” that was designed specifically for the evaluation of this type of literature ( Arcos *et al.*, 2021).

### Publication selection criteria, search procedure and technique

The literature has been selected considering the following aims of disinformation, which might be mentioned by potential research articles on the above topics: confusing the target; deceive the target; increasing uncertainty of the receiver about facts; information overload of the receiver; persuasion towards a partisan political option; sowing mistrust against government authorities or institutions; sowing mistrust against particular communities (example: immigrants); reflexive control or perception management.

Considering these keywords, to carry out the searches, search equations have been developed that include combinations of general keywords with other specific ones, to retrieve the most relevant recent literature, obtaining thosemost directly related to the objectives of the study.

“General word” AND (“Specific word 1” OR “Specific word 2” OR “Specific word 3” […]).

The main source were articles indexed in Scopus and published in the period of 1 ^st^ January 2014 to 17 January 2021 (keyword searches last run). This is based on the assumption that research on mis- and disinformation, also as part of hybrid threat activities, would have gained interest and momentum since the annexation of Crimea by the Russian Federation, allegations of Russian meddling in the U.S elections (2016), the Cambridge Analytica Case, the Salisbury attack, the publication by the EU of the Joint Framework for Countering Hybrid Threats (2016), and other allegations of foreign interference by the means of hostile information influencing ( Arcos, 2020).

For the retrieval of the search results, both the interrogation tools of the Web of Science and Scopus databases ( Birkle *et al.*, 2020) These databases have been selected because they are the ones that return the greatest number of results in this area thanks to their coverage ( Guerrero-Bote *et al.*, 2021). The Publish or Perish ( Harzing, 2010) software has been used. An alternative open source for retrieving and analysing the papers would be ScientoPy.

**Figure 2.**
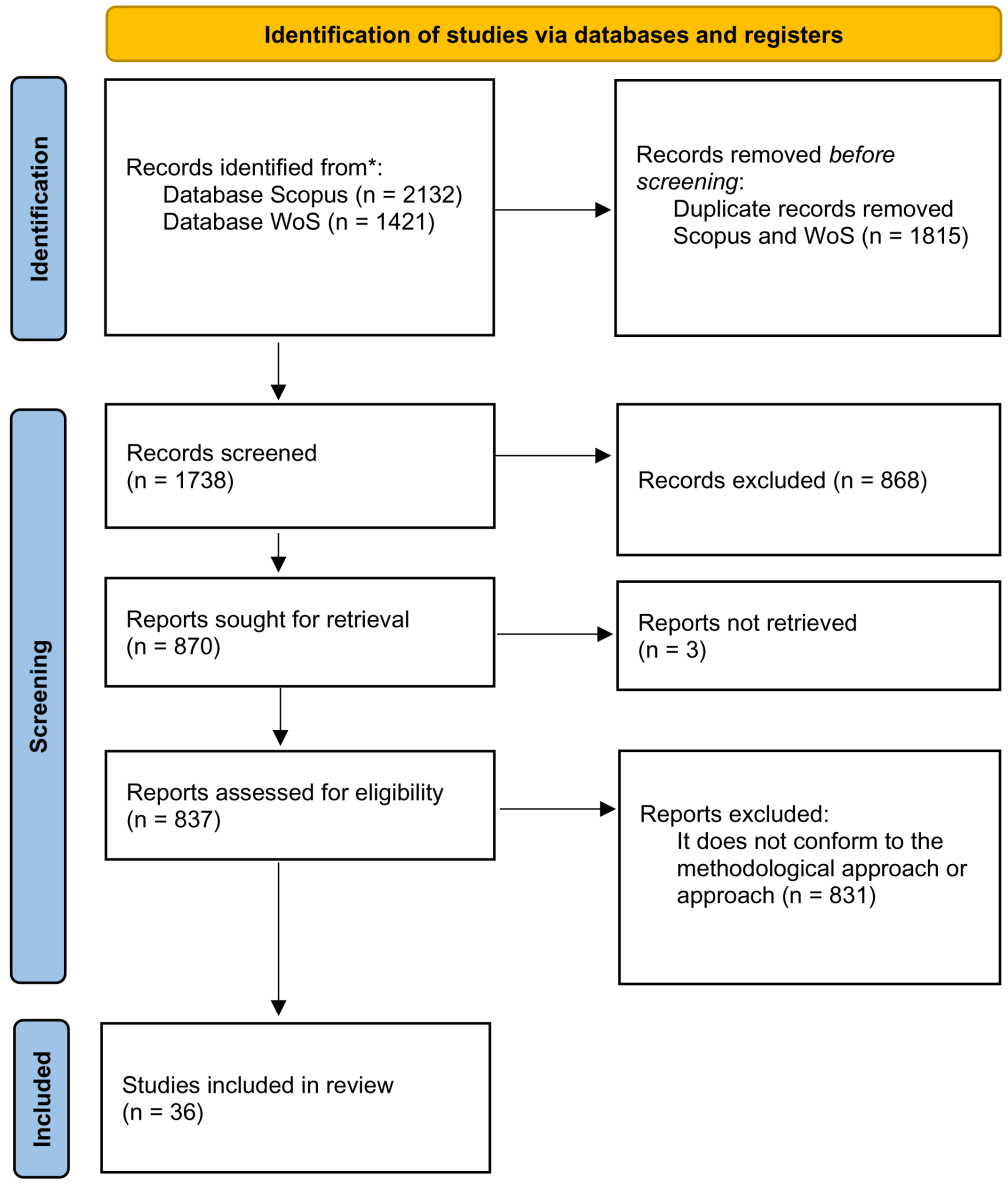
Application of the Preferred Reporting Items for Systematic Reviews and Meta-Analyses (PRISMA) framework and the EU-HYBNET Meta-Analysis Survey Instrument.

The flow diagram ( Figure 2) shows the details of the process followed for the Systematic review. In the eligibility process, results that are not related to the study approach (communication and security) have been eliminated. In this way, fields of a scientific-technical nature and other disciplines with which it is not possible to establish a relationship of interest with our approach have been left out.

Given that the six scientific journals of the International Association Communication are one of the main international references in this field, supplementary searches were performed in these journals with the last keyword search (=disinformation) conducted in April 2021. Specifically, the search was carried out in the following publications with these results: Journal of Communication (N=47); Communication Theory (N=10); Communication, Culture and Critique (N=9); Journal of Computer-Mediated Communication (N=9); and Human Communication Research (N=6) When these results were compared with those obtained in the WoS and Scopus searches, it was found that all these results had already been obtained in the previous process.

### Analytical evaluation of literature

Regarding content, the analytic instrument specifically analyses the potential use of samples in studies that make use of any of the following disinformation forms: apparently analytic pieces; fake news stories (hard news); opinion pieces, either with unintentional or intentional misleading information; opinionated retweets/posts distorting the meaning of the original news story; and public commentary of news stories published by digital news media (i.e. comments by readers). It also aims to detect literature on the topic that may capture the following practices: conspiracy theories; deepfakes (ai-generated photos, videos, sounds, texts); leak of apparently official documents; manipulation of images and other audiovisual materials (photoshopped-like edited photos and videos); micro-targeting; and revisionism of history strategy/tactics.

As for fact-checking and debunking, the meta-analysis instrument asks whether the effectiveness of fact-checking/debunking in target audiences is taken at face value, or if the study conducts an evaluation of its impact (beliefs, attitudes, behaviour) in target audiences. It also checks if there is any metrics employed, or an evaluation technique used to evaluate the impact of fact-checking or debunking, and what message strategies and tools are employed when debunking. A specific question on the employment of inoculation strategies against mis- and disinformation was also included.

## Results

### Findings of SLR analysis

Most of the studies have fact-checking effectiveness as the object of the study (n=12), followed by debunking effectiveness (n=9) and misinformation effects and/or impact (n=6). There are few studies that address disinformation and misinformation effects and/or impact (n=5) or disinformation as part of hybrid threats (n = 4) ( Arcos *et al.*, 2021b).

**Figure 3.**
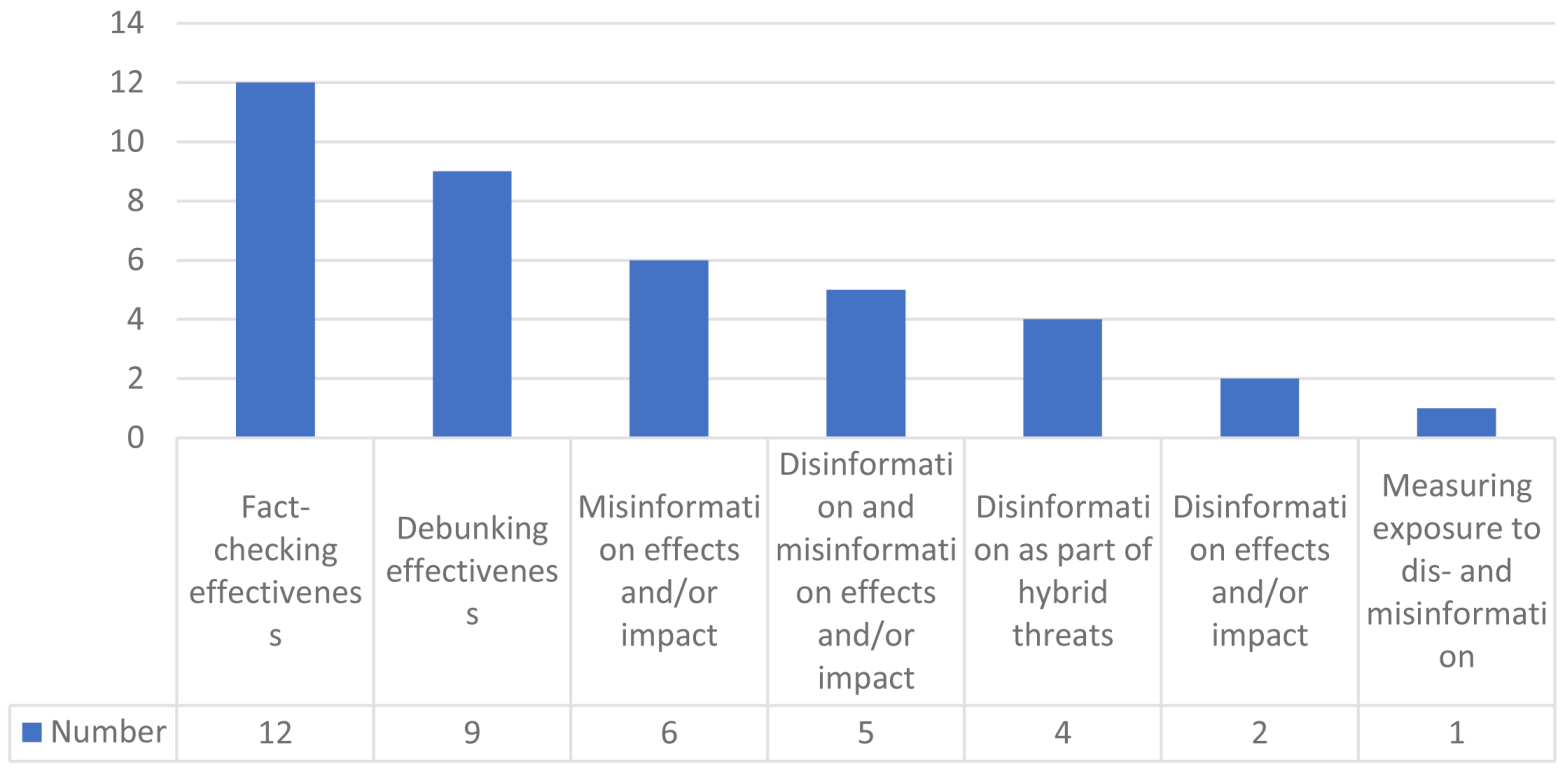
Distribution of investigations according to the object of study.

Figure 3 shows the complete distribution of investigations according to the object of study.

**Figure 4.**
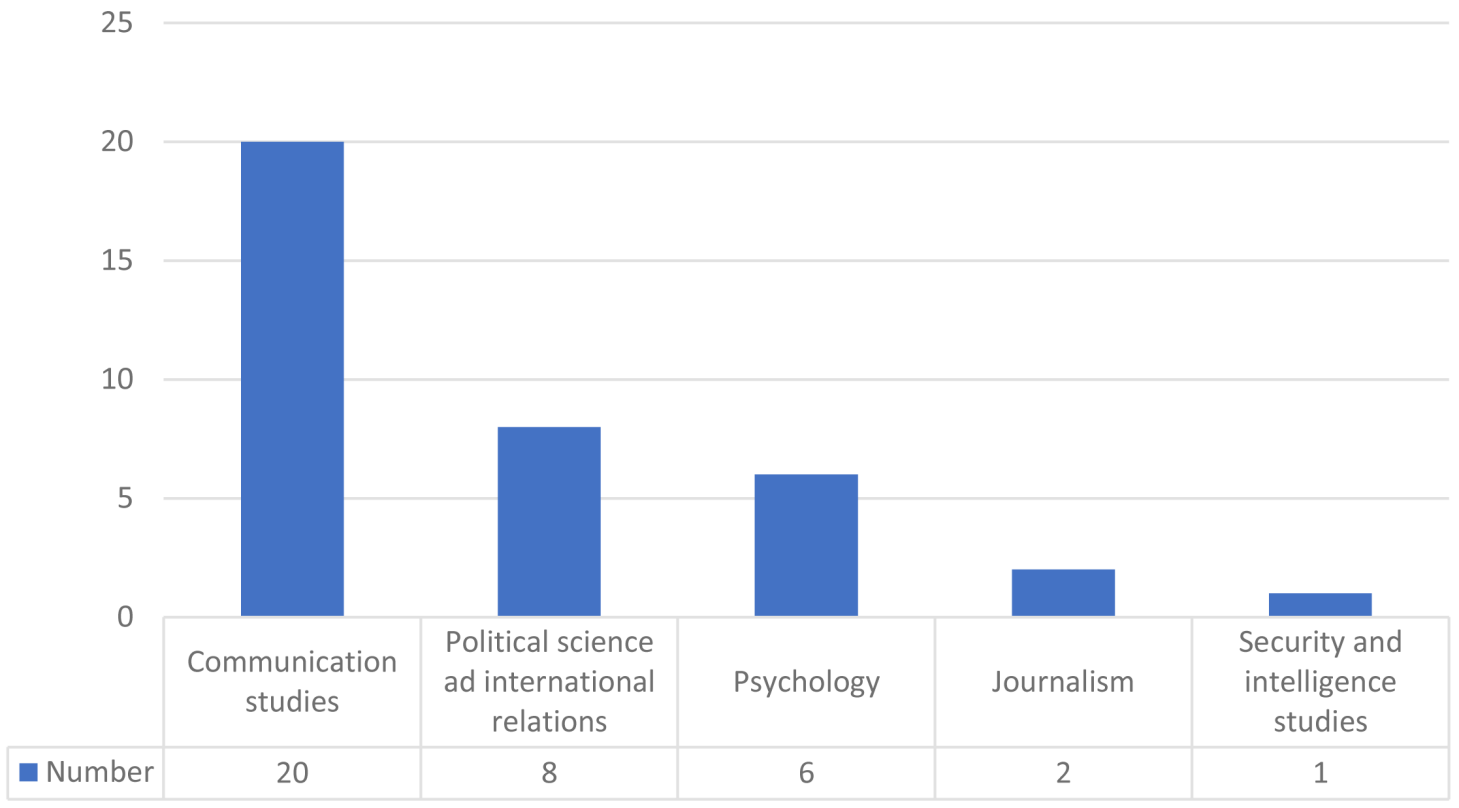
Academic discipline or field of study.

Most of the analysed works correspond to the academic discipline of communication studies, followed by political science and international relations and psychology ( Figure 4). 

The studies are fundamentally analytical (n = 18) and explanatory (n = 6). Few cases make a comparative or descriptive investigation (n = 3). The main information/data gathering technique used is experiments (n=19) and the collection of documents or audio-visual/digital media materials (i.e. tweets, posts, YouTube videos, WhatsApp messages) (n=13).

The kind of data obtained mainly is qualitative (n=18), quantitative (n=12) and experimental (n=9), with several investigations simultaneously applying more than one technique.

Most of the investigations declare the population of the study carried out (n = 27). Of these, most are probabilistic, some at the national level (United States population, UK-based participants, German population, Dutch nationally representative sample) others at the state level (general education course at Pennsylvania State University, general education course at University of Connecticut with a mean age of 19) or sectoral (Amazon Mechanical Turk [MTurk]).

**Figure 5.**
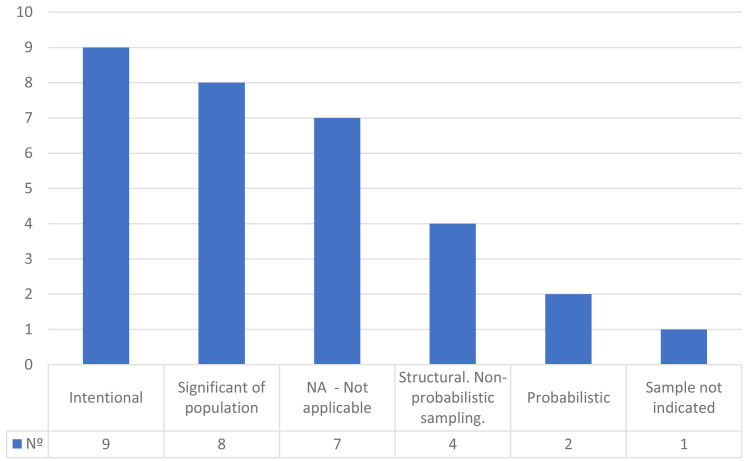
Classification of the studies according to the type of sample used.

There are also several studies that use convenience sampling, which is applied to cases with students (undergraduate students at a political science school in a large private university in France; undergraduate students of Communication; undergraduate students at a large university in the Midwestern United States; Twitter and other platform users). Figure 5 shows the classification of the studies according to the type of sample used.

A third of the studies (n = 11) have sample sizes greater than 500, eight between 201 and 500, and five between 101 and 200.

The theories used as a reference come mainly from cognitive science, social psychology, and communication. The main referenced theory is that of the Inoculation theory ( McGuire, 1964) (n = 6) as a protection system against persuasion or influence. Other theories or analytical frameworks used are the Attitudinal Filter of Motivated Reasoning, the Affective Intelligence Theory (AIT), the Epistemic Political Efficacy (EPE), the propaganda theories ( Chomsky & Herman, 1988; Lasswell, 1927) and the persuasion knowledge model ( Friestad & Wright, 1994), the social network theory ( Burt, 2005; Coleman, 1988) and intergroup competition theory ( Tajfel & Turner, 1979), among others.

The analysis techniques used are mainly quantitative (n=29). Although there is descriptive analysis, most are exploratory, inferential, and casual in nature. The following bivariate and multivariate analysis techniques are used: analysis of variance (ANOVA), multiple variance analysis and multiple regression analysis.

Six investigations refer to mis- or disinformation as part of hybrid threats/influence operations or information warfare.

The articles have a neutral approach to dis- misinformation in nine cases, but in 21 the analysis considers the political dimension, including causes and intentions. In these cases, they mention state actors (i.e. Russia, China, Iran, North Korea...) (n=10) and non-state actors linked or aligned with foreign governments (n=6)

Of the sample studied, 20 investigations analyse regional case studies, of which the majority (n = 16) focus on the United States, with unique cases for Germany, the Netherlands, or the United Kingdom.

The articles mention or analyse the following aims of disinformation, as indicated in Table 1.

**Table 1.** Aims of disinformation.

Aims	Frequency
Increasing uncertainty of the receiver about facts	11
Persuasion towards a partisan political option	10
Deceive the target	10
Sowing mistrust against government authorities or institutions	9
Confusing the target	6
Sowing mistrust against communities (example: immigrants)	4
Information overload of the receiver	1
Reflexive control or perception management	1

Other articles refer to issues like how the influence of audiences can be harmful to democracy, the cognitive and emotional effects of populist communication, the persuasion theories and the state propaganda, the specific mention of Russian propaganda in the context of Ukrainian war, or how the online political information with inaccurate beliefs harms democracies.

Table 2 shows the strategies analysed in the articles.

**Table 2.** Strategies analysed.

Strategies	Frequency
Conspiracy theories	9
Deepfakes (AI-generated photos, videos, sounds, texts)	3
Leak of apparently official documents	1
Manipulation of images and other audio-visual materials (Photoshopped-like edited photos and videos)	4
Micro-targeting	3
Revisionism of history	1

In other cases, it refers to public trust and influence on decision-making, the manipulation of comments spread by bots, the manipulation of websites logos and design, or the manipulation carried out from political parties.

**Figure 6.**
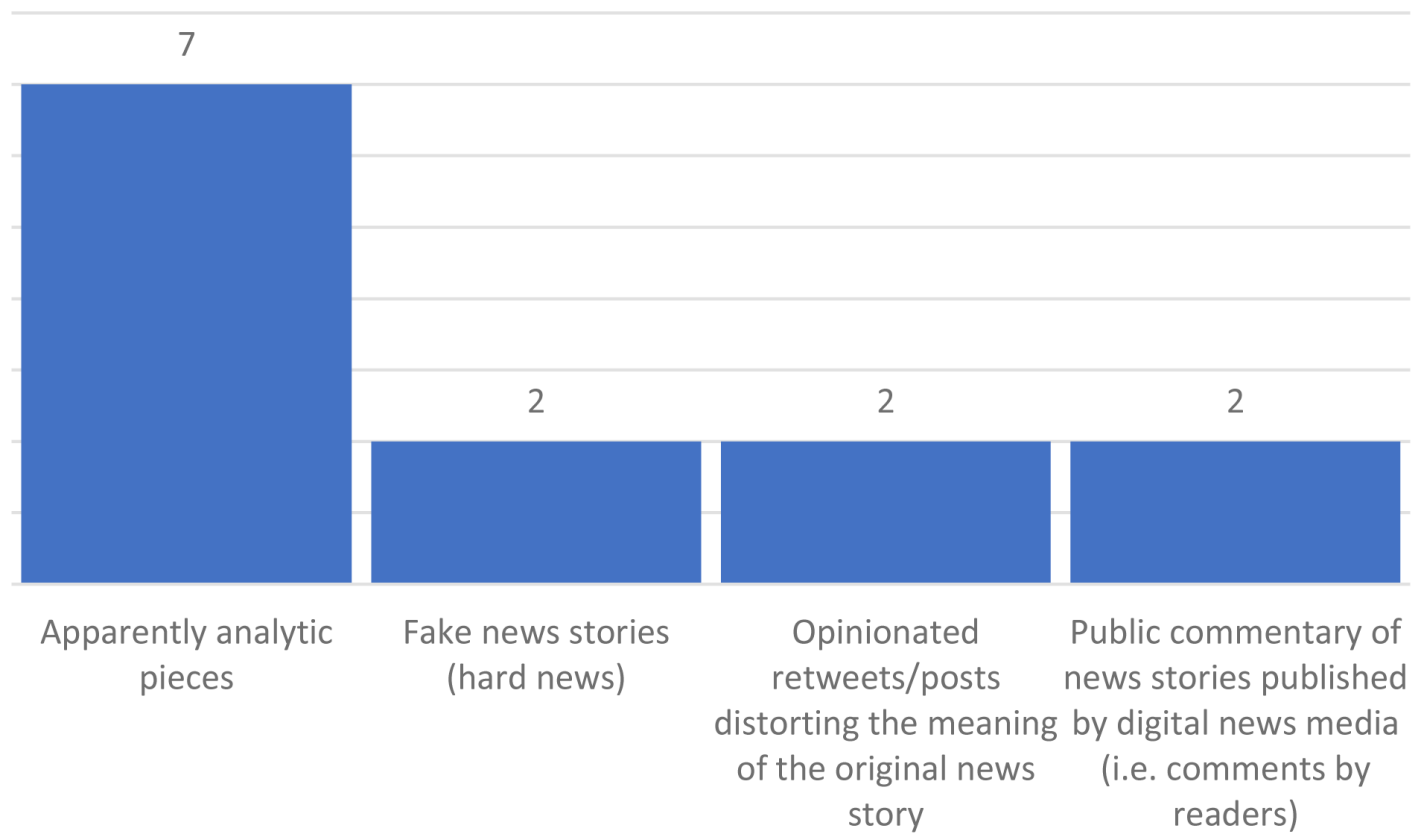
Types of documents analysed.

Regarding the types of documents that are analysed in the sample, the distribution is as follows in Figure 6.

Other documents are also mentioned, such as news article-like texts, soft news, memes, and news stories, deepfakes, real news and fake news from Snopes, posts from Twitter, Facebook, and Reddit, manipulated tweets from citizens, news agencies or fact-checking, native ads published by real media and fabricated headlines, among others.

Most of the research (n=24) adopts strategies of both emotional and rational persuasion together. In three cases only an emotional persuasion approach is used and in five only rational persuasion.

There are few references to the authenticity of the sources. In 15 cases no reference is made, in two it is attributed to bots, in another two to declared and official sources, and in another two to fake people. Other sources mentioned are fictional news organizations, credible media, alleged legitimate sources, trolls, news agency, real news, or fake websites.

**Figure 7.**
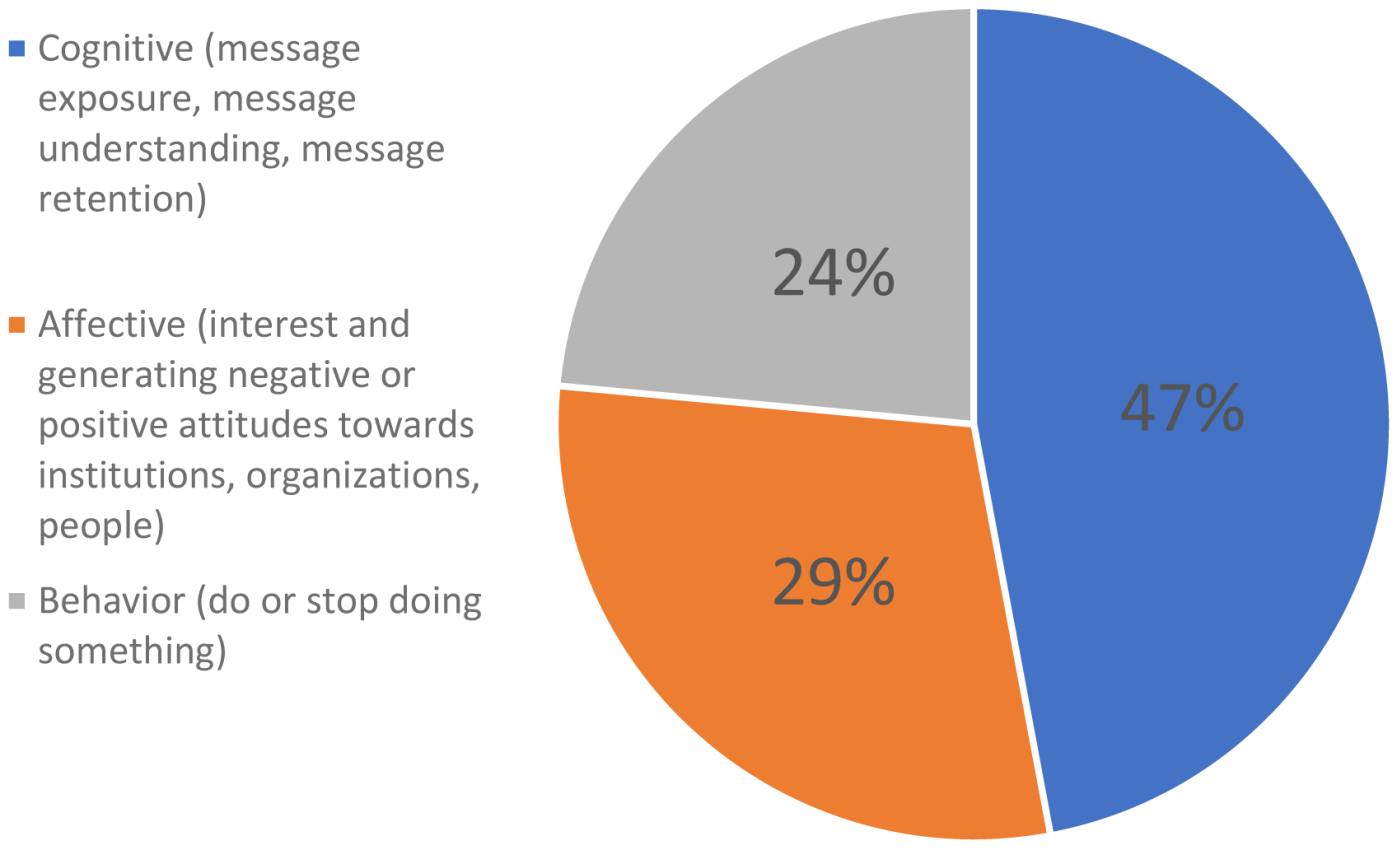
Effects reported of mis-and disinformation.

The following figure ( Figure 7) shows the effects reported about mis-and disinformation.

In eight cases a metric or an evaluation technique has been employed to evaluate the impact of mis- and disinformation. The techniques cited are, among others, Johnson & Slovic’s (1995) validated measurement model; Appelman and Sundar’s scale for measuring message credibility in the context of news; Measured by a finger tapping task (MTS or Maximum Tapping Speed) and expected MTS (self-estimated by the participants); Cohen’s D (measure of effect size); and metrics to assess the influence users’ message credibility and the effects like the scales used by Flanagin & Metzger (2007) and Zaichkowsky (1994).

There are 19 articles focused on the effectiveness of fact-checking or debunking. Half of the articles conduct an evaluation of the impact (beliefs, attitudes, behaviour) of fact-checking or debunking in target audiences. Of these, 14 focus on the effectiveness of fact-checking and/or debunking, and the other two studies conduct an evaluation of the impact. The first assesses the effect of warning labels (Facebook´s disputed by 3rd party fact-checkers) on perceptions of fake news accuracy, and the second develops an experiment to assess the accuracy, usefulness, tone and accepting claims of negative advertisements

Only one article reported a particular metric employed, or an evaluation technique used, to evaluate the impact of fact-checking or debunking. The authors develop a procedural news knowledge (PNK) to measure knowledge about the practices of institutions that produce news, editorial procedures that generate content, and distinctions between news gathering and advocacy. The interest of this instrument lies in the hypothesis formulated by the author that identified a relationship between the PNK and the resistance to disinformation.

Although there are nine articles that refer to the use of debunking, only two indicate what strategies or techniques are used. In one case, reference is made to inoculation (fact- vs. logic-based) messages, and in another to metainoculation messages, explaining how inoculation treatments work.

Seven articles have mentioned references to inoculation against dis- and misinformation. In some cases, to highlight its positive effects when facing disinformation that is incongruent with people’s prior beliefs, in others to explain its applications through the development of an agent-based model or as a general framework for research, referring to basic sources of this model ( Cook *et al.*, 2017; Lazarus, 1991; Lazarus, 1994; Roozenbeek & Van der Linden, 2018; Roozenbeek & Van der Linden, 2019) Finally, seven articles make reference to the backfire effect, highlighting that this effect is a subtype of confirmation bias.

### Findings of qualitative analysis

***Disinformation effects.*** Zach Bastick (2021) reported results from an experiment (N=233 participants; undergraduate students at a political science school in France) aimed at exploring the research question “Can fake news modify the unconscious behaviour of individuals?”, and consisting on deploying a finger tapping test “in which participants were asked to tap a single finger on a computer key at their maximum speed before and after being exposed to a fake news stimulus whose emotional valence was intended to alter their tapping speed” ( Bastick, 2021, p. 3), and found that “even a brief (under 5-min), one-time exposure to a fake news article can modify the unconscious behaviour of individuals” (p. 6).

Thorson (2015) has studied what she calls the “belief echo” through experiments comparing “political attitudes among those who are exposed to corrected misinformation and those who are not” and found that “exposure to a piece of misinformation can shape a person’s attitudes even though she recognizes it is false” (p. 1–2). Thorson contends that fact-checkers “operate under the implicit assumption that the correction will eliminate the misinformation’s effect on attitudes” but “the existence of belief echoes shows that this assumption is wrong” (p. 17).

Zerback *et al*. (2020) examined psychological effects of astroturfing (a strategy consisting of creating a false impression that specific opinions have a widespread support using websites, bots, and sockpupetting) in the context of Russian digital information influence, exposing over 2300 subjects to pro-Russian online astroturfing comments in Germany (p.1). They found “that astroturfing comments can indeed alter recipients’ opinions, and increase uncertainty, even when subjects are inoculated before exposure” (ibid.).

On the combination of deepfakes and political micro-targeting, Dobber *et al.* (2021) found through an experiment that “a deepfake meant to discredit a political candidate negatively affects people’s attitudes toward the depicted politician” but did not “negatively affects people’s attitudes toward the politician’s party”. Namely, that political micro-targeting is a potential amplifier of the deepfake “but only for a much smaller portion of the sample than we expected” (p. 82–84). Dobber *et al.* highlight that their findings differ from other existing studies and particularly from those of Bail *et al.* (2020) that found no evidence that interactions with the Russian Internet Research Agency (IRA) accounts “substantially impacted 6 distinctive measures of political attitudes and behaviours” (p.243) but that their “findings suggest that Russian trolls might have failed to sow discord because they mostly interacted with those who were already highly polarized” (ibid.). On IRA Twitter accounts, Zhang *et al.* (2021) highlighted that very successful accounts “like @TEN_GOP transformed themselves from average Twitter users with a few hundred followers in late 2015 to microcelebrities commanding over one hundred thousand followers before their discovery in September 2017” ( Zhang *et al.*, 2021, p. 2). These authors explain how this larger following facilitates impact, “Integral to this success was the accounts’ large Twitter followings, which (a) facilitated the direct dissemination of strategic messages by serving as their audiences; (b) functioned as a set of individuals likely to amplify their messages through retweeting and other means; and (c) provided visible engagement metrics that looked to other observers, including journalists, like evidence of authority and authenticity” (p. 2). 

Zhang *et al.* discussed the vulnerability to disinformation enabled by the interconnected media system, characterized as hybrid and asymmetric, political polarization, and the logic of attention economy within the media, highlighting that fact-checking/flagging, mis- and disinformation content, gatekeeping/moderation, and different literacies (i.e., media, digital) “only provide part of the answer” (p. 21).

Continuing with the activies of the Russian IRA, Lukito (2020) explored how its disinformation campaigns were “temporally coordinated through three social media platforms, Facebook, Twitter, and Reddit” (p. 249). In the time series analysis conducted during the period 2015–2017, the author found “that IRA may have used Reddit to test prototype messages prior to posting them on Twitter within a one-week lag.” However, they did not find a “relationship with the Facebook ads paid for by the IRA.” In addition, Twitter's preference over other platforms is evident, which can be explained “because of Twitter's unique position in the journalistic profession ( McGregor & Molyneux, 2018), tweets may have been shared in news stories” and “the platform’s reliance on short messages, which were easier and faster to produce.” (p. 249). Lukito highlights an important aspect of the modus operandi of disinformation campaigns that can be inferred for the study: that hybrid threat actors are, and particularly Russia, is “taking advantage of the multi-platform digital ecology to test and deliver message across different social media”.

Hjorth & Adler-Nielssen (2019) investigated the research question “How does ideology condition potential exposure to online, pro-Russian disinformation?” through a case-study focused on the communication flows in Twitter (U.S-based users) related to the crash of the MH17 aircraft over eastern Ukraine, and found that “ideologically conservative users are significantly more likely to follow disinformation accounts, compared to liberal users”, with the term “pro-Russian” meaning a standpoint “supportive of the current Putin regime and its political interests” ( Hjorth & Adler-Nielssen, 2019, p.169–170). The logic underlying their research is that there is a need for more research focused on the reach of pro-Kremlin disinformation, that is to say, who and how much is the audience in Western societies, so that this can enable to produce an estimate of the potential impact and outcomes of disinformation.

One other very relevant strand of research is related to the Truth-Default Theory ( Levine, 2014) whose “key idea is that when humans communicate with other humans, we tend to operate on a default presumption that what the other person says is basically honest” (p. 378). For considering the possibility of deception humans require “some trigger event that kicks a person out of their natural truth-default” or being prompted to do so ( Clare & Levine, 2019, p. 2). Hence, if communication content is presumed to be truthful by default, one of the consequences in the context of social media consumption is that users are vulnerable “to fake news, far-fetched conspiracy theories, and disinformation campaigns” and that attempts to decrease the impact of truth-default through increased vigilance “chronic suspicion is likely to spill over into increased scepticism of legitimate information sources” ( Clare & Levine, 2019, p. 20). On the effects and mechanisms of multimodal disinformation and rebuttals disseminated by social media in the context on school shootings and refugees, Hameleers *et al.* (2020) determined through an online experiment that including images to disinformation “significantly increases the perceived credibility of the message” (p.294). However, this effect is only relevant when disinformation is directed against refugees for being involved in terrorism (H1). The experiment also found no substantial differences in terms of credibility between the disinformation coming from a news source than ordinary citizens (H2).

The experiment found evidence that “perceived credibility of disinformation significantly decreases when any type of corrective information is presented after the disinformation”. (H3) (Hameleers *et al.*, p. 294). The results related to multimodal or textual fact-checkers do not provide the expected effects: “Multimodal fact-checkers that refute multimodal disinformation are not more effective than textual fact-checkers and in turn, multimodal fact-checkers are not significantly more effective in refuting textual than multimodal disinformation.” (H4) (p. 295).

On the psychological bias related to exposition to media, Van der Linden *et al.* (2020) describe what they call ‘fake news effect’, as the tendency for partisans “to use the term ‘fake news’ to discredit non-partisans media sources”. An experiment conducted concluded that liberal outlets (e.g., CNN) are “described as ‘fake news’ by conservatives and, in turn, conservative outlets (e.g. Fox News) are described as ‘fake news’ by liberals”. In terms of trustworthiness, “moderates and especially conservatives are less likely to trust the mainstream media than liberals” (p. 465). Also, the authors found evidence that” liberals associated ‘fake news’ more with politics, whilst conservatives associated the term more with media” (p. 464).

Regarding the effects of disinformation on terrorism, Piazza (2021) has studied the contribution of social media disinformation to domestic terrorism, theorizing and testing whether “disinformation disseminated by political actors online through social media heightens political polarization within countries and that this, in turn, produces an environment where domestic terrorism is more likely to occur” (p. 1). Piazza’s study, based on a sample of 150 countries for the period 2000–2017, found that “countries featuring the propagation of disinformation online through social media by governments, political parties and foreign governments do experience higher subsequent levels of domestic terrorism” and that “the impact of disinformation online on domestic terrorism is mediated through increased political polarization” (p. 3).

***Resilience to disinformation.*** A key aspect of deterrence by denial efforts against disinformation is that building the resilience of society and counting with indicators that allow for measurement in this context is key to improve responses and build that resilience. Humprecht *et al.* (2020) have developed a theorical framework based on different dimensions, seven measurable indicators and corresponding framework indices (populism index, polarization index, media trust, shared media, strength of the public broadcasting service, social media index, market size) (p. 12). According to the study, “low levels of populist communication, low levels of societal polarization, high levels of trust in news media”, a strong public service broadcasting, “high levels of shared media use, small-size media markets, and lower levels of social media use provide better conditions for resilience” (p. 9). Southern European countries are identified in a polarized cluster and are likely to be vulnerable to online disinformation (p. 16).

***Fact-checking and debunking.*** In their meta-analysis on the effects of fact-checking, Walter *et al.* (2020) tested different hypotheses on the issue. Some highlights from their findings are that,

- “Pro-attitudinal fact-checking (i.e. debunking the oppose ideology) resulted in stronger effects” than “counter-attitudinal fact-checking (i.e. debunking personal ideology)” (p.361).

- “The efficacy of fact-checking tended to weaken with increased political sophistication” (meaning that knowledgeable individuals with partisan views tend to scrutinize fact-checking more)

- The use of visual scales of truth in fact-checking “appears to backfire and attenuate the correction of misinformation” (p. 364).

Chan *et al.* (2017) studied the efficacy of debunking, and among other findings reported that “the debunking effect was weaker when the debunking message simply labelled misinformation as incorrect rather than when it introduced corrective information” and “that using a more detailed debunking message was effective to discredit the misinformation” but “did not translate into reduced misinformation persistence” (p.13) but was associated with greater misinformation persistence.

Pennycook *et al.* (2020) have discussed on the effects derived from using warning tags attached to the headlines of news stories, as a practice to counter political misinformation. Beside discussing earlier literature reporting backfire effects derived from using warnings when individuals are already biased against content that challenges their political beliefs (p.2), even their study does not explicitly mention foreign disinformation, but the consequences of what these authors called the “implied truth effect” still may be relevant for institutions and practitioners engaged in countering disinformation. According to these authors, “the implication of the absence of a warning is ambiguous: does the lack of a warning simply mean that the headline in question has not yet been checked, or does it imply that the headline has been verified (which should lead to an increase in perceived accuracy)? To the extent that people draw the latter inference, tagging some false news headlines with warnings will have the unintended side effect of causing untagged headlines to be viewed as more accurate” ( Pennycook *et al.*, 2020, p. 2). Findings reported from their experimental studies suggest that “putting warnings on blatantly false content may make other kinds of (potentially more insidious) misinformation seem more accurate” (p. 12).

Garrett & Poulsen (2019) conducted an experiment to identify the more effective fact-checking flagging strategy. Through testing three different kind of flags (tested fact-checker flags, peer-generated flags, and a flag indicating that the publisher self-identified as a source of humour) the authors concluded that “self-identified humour flag tended to be more effective than the other two types of flags, reducing beliefs and sharing intentions”. On the other hand, the authors found no evidence of benefits associated with warnings form fact checkers or peers.

Margolin, Hannak and Weber’s (2018) study on the effects of political fact-checking on Twitter found that “underlying social structure is an important factor in the correction of misinformation”. This means that “when the people involved in the correction have a mutual relationship, the correction is more likely to be accepted” (p. 202). The authors did not find differences in the correction of political and non-political rumours, in both cases the dynamics of the corrections are similar: “corrections from friends and followers are more likely to be accepted.” (p. 214)

Chung & Kim (2020) have studied, through an experiment, the role of fact-checking in deterring the sharing of fake news by individuals, instead of on the correcting role of fact-checking. They hypothesized and found supporting results for the hypothesis that “increased TPP (third-person perception) as a function of fact-checking (H2) will mediate the influence of fact-checking on social sharing intentions, such that those who viewed a news story with debunking fact-checking information will report weaker intentions to share the news on social media than those who viewed the news story without fact-checking information” (p. 5).

Banas & Miller (2013) examined the effects of inoculation ^i^ against conspiracy theories as well the effectiveness of meta-inoculations (inoculation applied to inoculation). As explained by these authors, conspiracy theories are challenging because “they defy the rational, logical, and reasoned approach exemplified by inoculation interventions” and because “conspiratorial arguments often employ circular reasoning, repetition of unproven premises, no falsifiable premises,” and other stratagems (p. 187). Through an experiment structured in three phases they found that “both the inoculation treatments induced more resistance than the control message, with the fact-based treatment being the most effective; and “that metainoculation treatments reduced the efficacy of the inoculation treatments.” (p. 184).

Of relevance for institutional communicators are findings from a study by Van der Bles *et al.* (2020) on “whether communicating epistemic uncertainty about facts across different topics (e.g., global warming, immigration), formats (verbal vs. numeric), and magnitudes (high vs. low) influences public trust.” (p.7672). Although the study was not focused on countering disinformation or on the attribution of specific activities to hostile actors, still it is very relevant from the perspective, for example, of how to communicate uncertainty by authorities or research organizations on the potential involvement of specific hostile actors in hybrid threats activities when you don’t have all the evidence at a given moment. Findings from the study suggest that it is key to be honest and transparent regarding the limitations of research and cognition, and communicating uncertainty with numeric probabilities instead of through linguistic markers of likelihood (which are very ambiguous). Findings from the experiment reported by Van der Bles are that "verbal quantifiers of uncertainty, however, do seem to decrease both perceived reliability of the numbers as well as the perceived trustworthiness of the source” (p. 7680).

## Discussion and conclusions

Our meta-analysis of literature focused on mis-/disinformation effects and fact-checking/debunking effects shows that what we may call the effects component of disinformation studies is an important subfield of research that is receiving increasing attention and producing relevant scholarship that provides evidence-based findings on the dynamics of online mis- and disinformation and on the effectiveness of fact-checking/debunking practices ( Feldman, 2017; Bernal-Triviño & Clares-Gavilán, 2019), what practices work better and the challenges derived from psychological factors, social factors, and the structure of the current media system of our digital age ( Salaverría *et al.*, 2020).

The SAL shows that, although the growth of these studies is recent, they are extensive investigations, with analytical and explanatory methodologies, using large samples (above 500 in many cases) and thorough experimental processes. However, most studies of this nature analyse regional case studies from the United States ( Elswah & Howard, 2020). In general, the analytical techniques used are mainly quantitative.

While some theories that emerged for gaining a better understanding of the effects of traditional media and news media organizations are still in place and can be useful for providing explanations and predictions, there is a need to update them in the light of the transformation that the production and distribution of information have experienced over the last few decades. Assessing the reach and impact of disinformation requires reliable data on audiences and their exposure to disinformation messages for a full understanding of its impact; these audiences can be simultaneously exposed to incoming information through multiple devices ( Valkenburg & Oliver, 2020). This has increased the complexity of producing audience studies that latter can be used in assessing the impact of disinformation.

Overall findings from the experiments reported in the literature suggest that disinformation produces effects on audiences, although the effects of disinformation depends on several factors, and pre-existing attitudes and beliefs play a very important role on the acceptance of disinformation content by individuals ( Ewoldsen & Rhodes, 2020). Studies show that correcting dis- and misinformation through fact-checking is not necessarily effective for all subjects, and that corrections that come from friends and those with a mutual relationship tend to be more effective.

Holders of partisan positions are not only more vulnerable to disinformation attacks consistent with their views, but also will likely be more resistant to debunking, unless the debunking message is consistent with their initial positions. Existing experiments on the effects of deepfakes combined with micro-targeting suggest that there is a potential amplification effect of micro-targeting, but still prior attitudes and beliefs will mediate on the effects.

Reports from experiments on inoculation effects against disinformation and conspiracy theories have found that inoculation can be a successful strategy, although disinformation actors can develop meta-inoculation practices and reduce the effectiveness of inoculation.

Our currently fragmented media ecosystem presents a challenge in countering disinformation, in part because fact-checkers and news organizations cannot debunk the number of mis- and disinformation messages spread across multiple platforms and do not have the time required to do so. Some sites and platforms can be used as laboratories to “test the waters” for customized polarizing, deceitful, and malicious content aimed at target audiences, disseminating disinformation through platforms where they can reach larger audiences and engage with journalists and with accounts with a high number of followers.

Assessment of the effects of disinformation and hostile activities in the information domain as part of hybrid threats should introduce the time perspective before extracting definitive conclusions; short term effects might not be the only effects to consider, since some activities might be part of the priming phase before the posterior full effects become apparent ( Giannopoulos *et al.*, 2021).

The periodic conduction of public opinion polls aimed at understanding individual’s perceptions of issues that have been part of the disinformation narratives of foreign actors is necessary for informing strategic communications efforts by governments and international institutions. The long-term impact of disinformation, hostile media content ( Jack, 2019), and the influence operations by foreign state actors in society is difficult to ascertain unless regular public opinion studies are conducted.

## Data availability

### Underlying data

Zenodo: Dataset. Responses to digital disinformation as part of hybrid threats: an evidence-based analysis on the effects of disinformation and the effectiveness of fact-checking/debunking. https://doi.org/10.5281/zenodo.5546503 ( Arcos *et al.*, 2021b)

This project contains the following underlying data:

● EU-HYBNET-Meta-Analysis-Dataset.csv. It contains the complete dataset including the reference of the evaluated titles and authors, and the data of the analyzed variables.

### Extended data

Zenodo: EU-HYBNET Meta-Analysis Survey Instrument for Evaluating the Effects of Disinformation and the Effectiveness of counter-responses. https://doi.org/10.5281/zenodo.4521174 ( Arcos *et al.*, 2021)

This project contains the following extended data:

● EU-HYBNET-Meta-Analysis.pdf. Analytical instrument for evaluating both scientific literature on the effects of mis- and disinformation and the effectiveness of fact-checking and debunking, as well as studies and reports developed by practitioners, institutions and think-tanks. It includes forty questions aimed systematically examining the evaluated documents.

### Reporting guidelines

Zenodo: PRISMA checklist for ‘Responses to digital disinformation as part of hybrid threats: an evidence-based analysis of the effects of disinformation and the effectiveness of fact-checking/debunking’ https://doi.org/10.5281/zenodo.5546521 ( Arcos *et al.*, 2021a).

Data are available under the terms of the Creative Commons Attribution 4.0 International license (CC-BY 4.0).
