# Supplementary material for: Responses to digital disinformation as part of hybrid threats: a systematic review on the effects of disinformation and the effectiveness of fact-checking/debunking
Source: Open Res Eur. 2022 Jan 13;2:8. [Version 1] doi: 10.12688/openreseurope.14088.1 (PMC10446021; doi:10.12688/openreseurope.14088.1)
Supplement: Peer review history

# Peer review: Reviewer response for version 1

**DOI:** 10.21956/openreseurope.15187.r28363

**Published:** 2022-01-26

**Authors:** Gordan Akrap

**Affiliations:** 1 Hybrid Warfare Research Institute, Zagreb, Croatia

The article is very well organized and proves its publication and further dissemination to the academic society. It shows the ways in which we need to make further researches in order to develop different residencies within the targeted states and societies. It shows some of the challenges that academic circles just as practitioners are facing and will be faced with in the future. It shows also that some of the impacts that article is trying to identify and describe need to be further developed in order to identify and define early warning signals that can be helpful to understand and recognize hybrid threats before they appear.

 There are a few things that need to be mentioned. They do not affect negatively the article. They might be used as a part of further researches. In the abstract, the authors wrote that "Disinformation can also be part of hybrid threats." Disinformation is always part of the influence operations that are one of the most significant vectors of attack in hybrid threats. That is also mentioned in the article in several places. Second thing is that this article argues about foreign influence/interference. It is important to say that those activities can be conducted by internal actors also that might (or they are) connected with some foreign actors.

 It will be excellent if authors can, using the same methodology, make an analysis of articles published in DOAJ or ERIH PLUS and compare results from those two analyses.

 The main objective of the article needs to be widened with, what is mentioned in the article, the ability to be resilient against the malicious influence of the disinformation.

 There is one mistake in the article. On page 6, Figure 2, in box with text: "Reports assessed for eligibility" number 867 needs to be there (not number 837 as it is now).

 The article will be easier to understand to a wider audience if the sentences are a little bit shorter.

If applicable, is the statistical analysis and its interpretation appropriate?

Yes

Is the study design appropriate and is the work technically sound?

Yes

Is the work clearly and accurately presented and does it engage with the current literature?

Yes

Are the conclusions drawn adequately supported by the results?

Yes

Are sufficient details of methods and analysis provided to allow replication by others?

Yes

Are all the source data and materials underlying the results available?

Yes

Reviewer Expertise:

Information and communication sciences, Intelligence and security sciences, Strategic and crisis communication, Hybrid Warfare, Hybrid Threats, Influence operations

I confirm that I have read this submission and believe that I have an appropriate level of expertise to confirm that it is of an acceptable scientific standard.

# Peer review: Reviewer response for version 1

**DOI:** 10.21956/openreseurope.15187.r28362

**Published:** 2022-01-19

**Authors:** Ruxandra Buluc

**Affiliations:** 1 “Carol I” National Defence University, Bucharest, Romania

The article provides both a quantitative and qualitative analysis of recent scientific studies on the issues of disinformation, misinformation, strategies of producing disinformation and misinformation, and the effects that fact-checking and debunking can have on reducing the effects of disinformation and misinformation. 

 The authors employ two main methods of research: the Preferred Reporting Items for Systematic Reviews and Meta-Analyses (PRISMA) framework and a systematic literature review, whose application is clearly explained in the article.

 One observation is that the authors declare that the main objective of the article is:

 "to identify relevant findings from the existing literature that fall under the following categories (...) ● Disinformation effects and/or impact; ● Misinformation effects and/or impact; ● Disinformation and misinformation effects and/or impact; ● Disinformation as part of hybrid threats; ● Fact-checking effectiveness; ● Debunking effectiveness; ● Measuring exposure to dis- and misinformation".

 However, in the section entitled "Findings of qualitative analysis" they only address two of these categories "disinformation effects" and "fact-checking and debunking" and they introduce another category, not mentioned previously, namely "resilience to disinformation". More explanation could be provided for the reason why the categories are regrouped, why certain categories such as "disinformation as part of hybrid threats" is not approached in the qualitative analysis, and why they felt the need to add the category resilience to disinformation only at this stage in their research.

If applicable, is the statistical analysis and its interpretation appropriate?

Yes

Is the study design appropriate and is the work technically sound?

Yes

Is the work clearly and accurately presented and does it engage with the current literature?

Yes

Are the conclusions drawn adequately supported by the results?

Yes

Are sufficient details of methods and analysis provided to allow replication by others?

Yes

Are all the source data and materials underlying the results available?

Yes

Reviewer Expertise:

Strategic communication, disinformation campaigns, security culture, intelligence analysis

I confirm that I have read this submission and believe that I have an appropriate level of expertise to confirm that it is of an acceptable scientific standard.
